# Diabetic patient assessment of chronic illness care using PACIC+

**DOI:** 10.1186/s12913-020-05400-5

**Published:** 2020-06-16

**Authors:** Maria Malliarou, Christina Desikou, Eleni Lahana, Styliani Kotrotsiou, Theodosios Paralikas, Athanasios Nikolentzos, Evangelia Kotrotsiou, Pavlos Sarafis

**Affiliations:** 1 Department of Nursing, University of Thessaly, Perifreiakh Odos Larisas, Trikalon, TK 41110 Larisa, Greece; 2 General Public Hospital of Volos “Achillopouleio”, Athanasaki 3, TK 38222 Volos, Greece; 3 University of Thessaly, Perifreiakh Odos Larisas, Trikalon, TK 41110 Larisa, Greece; 4 Hellenic Open University, Parodos Artistotelous 18, TK 26335 Patras, Greece; 5 Department of Nursing, Cyprus University of Technology, 30 Archbishop Street, 3036 Limassol, Cyprus

**Keywords:** Chronic illness, Diabetes, Quality of life, Chronic care model, CES-D, SF-36

## Abstract

**Background:**

The Patient Assessment of Chronic Illness Care plus is used in order to assess whether provided care is congruent with the Chronic Care Model, according to patients. The purpose of this study was to correlate PACIC+ and the revised 5As “ask, advise, agree, assist and arrange” scoring of a sample of DM patients, with their QoL, depressive symptomatology, demographic and disease characteristics, self-management behaviours of healthy eating and physical activity.

**Methods:**

This is a cross-sectional study where data were collected between January and April 2018 by using three questionnaires (PACIC+, SF-36, CES-D) from a sample of 90 DM patients treated at a Public General Hospital of Central Greece. Anonymous self-completed questionnaires were used to collect the data. Data was processed in the Statistical Package for the Social Sciences (SPSS).

**Results:**

The mean age of the participants with DM was 52.8 years (SD = 21.2 years), with cardiovascular disease and arterial hypertension scoring as the most frequently reporting chronic comorbidities. The healthcare received by DM patients has been correlated with their QoL. More specifically SF – 36 and PACIC+ scale scores showed a positive and low correlation in several subscales. The total score of PACIC+ scale as well as the Patient activation score were increased in higher scores of vitality (*p* = 0.034 & *p* = 0.028 respectively), hence both scores correlate significantly with latter. In addition, Delivery System / Practice Design score was increased in higher scores of mental health (*p* = 0.01) and MCS (*p* = 0.03).

**Conclusions:**

The shift from hospital care focusing on the disease to a more patient-oriented approach puts forward a dynamic holistic approach to chronic diseases and the reduction of their impact. Finding evidence-based and effective strategies to promote health, prevent and manage chronic diseases such as diabetes mellitus is deemed to be crucial and necessary. PACIC+, which is a tool of a patient-level assessment of CCM implementation, can be used by countries which intend to apply changes in the way their health systems provide chronic care and specifically wish to improve the quality of chronic disease care and the QoL of their patients.

## Background

Diabetes mellitus (DM) is a group of metabolic disorders characterized by high blood sugar level over a prolonged period of time [1, 2]. The most common forms of DM are Type I (absolute insulin deficiency due to destruction of insulin-producing β-cells) and Type II (secretion and/or insulin action disorder). Other specific types of diabetes are rare specific cases of secondary presentation [3] of the disease and gestational diabetes that often subsides after childbirth.

The global diabetes prevalence in 2019 was estimated to be 9.3% (463 million people), rising to 10.2% (578 million) by 2030 and 10.9% (700 million) by 2045 [4]. Despite the limited available data on the prevalence of DM in Greece, relatively recent studies summarize the rate of drug-treated DM in 7% [5].

The autogenous complications in the DM, apart from vital organs, burden the mental health and social life of the sufferers and their relatives [6], causing further difficulties in achieving the goals and quality of their lives [7]. Diabetic complications often result in motor disabilities with depressive symptoms [8], and the chronicity of the condition frustrates the individual, as it weakens its strength and reduces its mental strength, resulting in adverse health effects, and non compliance to therapy [9, 10]. On the other hand, adaptation to illness is often accompanied by a variety of negative emotional reactions such as anger, guilt, disappointment, denial and loneliness [9, 11].

Diabetes, as well as cardiovascular diseases, asthma and hypertension is classified under chronic diseases [12–14]. Therefore, diabetes management requires integrated care and advanced access to it. Dynamic empowerment with a specific focus on maintaining a person’s health, prevention and proper management without relapse to chronic diseases, is considered to be necessary [15–17].

In order to accelerate this kind of benefit transition for more than 133 million people living with a chronic disease [18], which represented 70% of the reported deaths worldwide in 1998, the Improving Chronic Illness care program created the Chronic Care Model which aimed at empowering health care improvements at the community, organization, practice and patient levels [19, 20].

The Chronic Care Model introduced well-documented concepts of change, combining the promotion of productive interactions between well-informed patients who take an active role in their care and resource management under the expert guidance of health care providers [21–23]. The Chronic Care Model (CCM) is promoted as a guide to improve chronic care and to realise patient-centred care in which problems such as fragmentation, guideline non-adherence, and restricted self-management are limited [22]. Patient Assessment of Chronic Illness Care (PACIC+) questionnaire has been developed, translated and applied internationally to patients with chronic diseases such as diabetes, hypertension, cardiovascular diseases and asthma [21, 24–33]. The Patient Assessment of Chronic Illness Care (PACIC+) measures the extent of alignment of chronic care with the CCM, and measures patients’ chronic care experience. The model promotes the reconstruction of health care within the aim of continuous, coordinated and multi-sided care through health system [22, 34].

Poor management and control of diabetes often leads to poor disease outcomes, including impaired health-related quality of life (HRQL) and health status [35–37]. A tool that assesses the physical, psychological and social well-being of the individuals offers a comprehensive picture of the impact of the disease on the individual, as opposed to physiological and clinical examinations, which usually only detect health problems [35]. In essence, HRQL’s assessment completes the clinical evaluation, adding the important element of the subjective assessment of his/her physical and psychosocial health by the patient himself/herself. Therefore, the wide range of information received by a HRQL assessment can contribute (a) to a sound and in-depth knowledge of the physical and psychosocial effects of the disease, (b) to a better understanding of the causes that patients react differently to the same disease, and (c) to planning and implementing clinical interventions and services to address the health effects of illnesses [38–40].

Various QoL assessment questionnaires have been developed for the DM in particular, but they are either too extensive and lack in application specificity [18], or are not susceptible to the generalization of their findings. The Short Form- 36 (SF-36) questionnaire, which is a general questionnaire, evaluates the physical and mental health of the individual, by using 8 scales: physical functioning (PF), role physical (RP), bodily pain (BP), general health (GH), vitality (VT), social functioning (SF), role emotional (RE), and mental health (MH). SF-36 is characterized by reliability and validity and is one of the most widely used tools for measuring QoL [41].

The SF-36 is widely accepted as one of the most commonly used general health measure in QoL with an obvious degree of reliability and validity. One of its most important features is that it provides age-matched and sex-matched population normative data [42].

Regarding the diabetes-specific measures, such as ADDQoL and DQoL and ADS, the latter is known to acquire valuable traits such as validity and comprehensiveness in terms of scoring. The first two diabetes specific measures, ADDQoL and DQoL, have been tested numerous times and present an increased degree of validity and reliability compared to ADS. However, issues in terms of feasibility constitute them unattractive for use in clinical settings. Finally, the DQoL includes several extra questions resulting in a rather lengthy form of questionnaire with enhanced complexity in terms of its opening questions that potentially could affect choices on the remaining items [42].

As far as CES-D scale is concerned its items are associated with depression and it has presented high degree of reliability when assessing the number, types and duration of depressive symptoms across racial, gender and age categories [43–45] with additional high internal consistency since Cronbach’s coefficients range from .85 to .90 across studies [43].

The purpose of this study was to correlate PACIC+ and the revised 5As “ask, advise, agree, assist and arrange” scoring for a sample of DM patients with their QoL, depressive symptomatology, demographic and disease characteristics, self-management behaviours of healthy eating and physical activity.

## Methods

The research questions of the study at hand are the following:

- How do PACIC and 5As scores correlate to *1*) patient characteristics, *2*) self-management behaviours of healthy eating and physical activity?
- How do PACIC and 5As scores correlate to patients with DM, QoL or depressive symptomatology.

### Participants

The study comprised of 90 adult patients treated at a Public General Hospital of the city of Volos in Central Greece who were recently diagnosed (the last 6 months) with type I and type II DM.

### Procedure

The study population consisted of a convenience sample of 90 patients with DM. The research was conducted between January and April 2018, in Volos Greece. Exclusion criteria included the inability of the patients to respond to the questionnaires due to the severity of their state of health. Patients with DM from the Intensive Care Unit, the Operating Room and the Emergency Department were excluded. Following the approval of the scientific council of the hospital, the self- completed questionnaires were distributed. All potential participants were instructed that the study was voluntary and confidential.

### Measures

The questionnaire included demographics, data on the disease of DM, and the scales of PACIC+, the CES-D and SF-36. PACIC+ is a reliable, psychometrically tested for Greek patients instrument to measure the chronic care management experiences of patients. It has five subscales: 1) Patient activation (3 items), 2) Delivery system design/Decision support (3 items), 3) Goal setting (5 items), 4) Problem solving/Contextual counselling (4 items), and 5) Follow-up/Coordination (5 items) [20, 21]. Six additional questions are included in this 5As model questionnaire to enhance the rating of its scales. 5As is a behavioral counseling model based on the scales of appraisal / appraise-advise-agree-help-organize and conform to the Chronic Care Model. Response categories in 20 questions range from 1 ‘almost never’ to 5 ‘almost always’, with higher scores indicating a higher extent to which patients received integrated care. The reliability was assessed and Cronbach alpha was found to be 0.913. In order to compare QoL of DM patients our research team used the SF-36 questionnaire.

The SF-36 comprises of 36 questions that measure eight dimensions of health: physical functioning (PF), role physical (RP), bodily pain (BP), general health (GH), vitality (VT), social functioning (SF), role emotional (RE), and mental health (MH). In addition to dimension scores, two summary scales (the Physical Components Summary [PCS] and the Mental Components Summary [MCS]) can be derived from the scales [46]. These two subscales with 8 items each, are scored from 0 to 100 with higher scores indicating higher levels of functioning. The reliability was assessed and Cronbach alpha was found to be 0.532.

As depression has been presented in several studies as a potential confounder factor of QoL and of satisfaction with care in most chronic disease patients, including DM patients, we used the CES-D scale in order to assess the depressive symptomatology of DM patients. Our research took also into account that depression has been widely presented as an independent predictor of mortality in many chronic diseases [43, 47, 48]. The CES-D scale is a short self-report scale designed to measure depressive symptomatology in the general population, including 20 questions trying to investigate depressed mood, loss of pleasure and interest, weight loss / decreased appetite, insomnia, psychomotor vigilance or deceleration, fatigue and decreased energy, low self-esteem and concentration disorder, including two additional questions specifically aimed at investigating suicidal behavior. CES-D total scores range from 0 to 60, with higher scores indicating more severe symptomatology. The reliability was tested by assessing the internal consistency which resulted in an overall Cronbach alpha of 0.887.

### Data analysis

Descriptive analysis was applied to describe the baseline characteristics of the study population. Continuous variables are presented as mean ± standard deviation, while qualitative variables as absolute and relative frequencies (%). Normal distribution of variables was tested with the Shapiro-Wilk test. Pearson chi squared and fisher exact test was used in correlating categorical variables. Pearson correlation coefficient was used for the association of score measurements. Tests were 2-sided and the statistically significant level was α = 0.05. Missing values were excluded from the analysis. Analysis conducted using SPSS v19.

## Results

The 90 subjects of the sample who suffered from diabetes mellitus (Type ΙΙ 64.4%) were 52.8 ± 21.2 years old. Table 1 presents the relative frequency (%) for two major types of diabetes together with other categorical data.

**Table 1:** Demographics of the sample

	***(N)***	***%***
Gender	(90)	
Men	(35)	38.9
Citizenship	(90)	
Greek	(80)	88.9
Marital status	(90)	
Married	(55)	61.1
Education	(86)	
Compulsory		39.5
Secondary		23.3
Higher		37.2
Employment Status	(85)	
Employed		49.4
Unemployed		15.3
Retired		35.3
Income	(86)	
**< 10.000 €**	**(47)**	**54.7**

Physical activity was low for 50% (*n* = 45) of participants and high for the remaining 50% (*n* = 45). Most of them are non-smokers 60% (*n* = 54), 18.9% (*n* = 17) are ex-smokers and 18.9% (*n* = 17) smokers. Associated diseases are reported in 38.9% (*n* = 35) of the participants, while 57.8% (*n* = 52) was on balanced diet. 61.1% (*n* = 55) of the participants were diagnosed with diabetes in a random checkup. 51.1% (*n* = 46) of them were treated with diet, 54.4% (*n* = 49) used insulin injections, 48.9% (*n* = 44) peros medication and 25.6% (*n* = 23) were treated with physical activity. PACIC+ scale scores were in similar level in all PACIC subscales (mean score range: 3.1–3.7). The mean summary score on the PACIC was 3.3 of a possible 5. 5as scoring: The mean for the overall 5As summary score was 3.1 of a possible 5. 27.2% of the participants had a mean score of CES-D 14.9 (SD ± 11.1). SF – 36 scale scores were in a wider range (mean score: 40.6–74).

Multiple linear regression (stepwise method) with dependent variable the PACIC+ scale and independent variables demographic characteristics and CES-D scale revealed that eating habits, nationality and depression score were found to correlate independently with PACIC score (Table 2). More specifically participants who had a healthy diet scored 0.46 units higher in PACIC+ indicating better care received compared to participants who did not have healthy diet. Participants with nationality other than Greek scored 0.57 units lower in PACIC+ than those with Greek nationality and those with more depressive symptoms scored lower in PACIC+.

**Table 2:** Multiple linear regression (stepwise method) (*N* = 90)

Multiple linear regression (stepwise method) with dependent variable the PACIC+ scale and independent variables demographic characteristics and CES-D scale (*N* = 90)				
		**Coeff.**^**a**^	**SE**^**b**^	**P**
Healthy diet	no (reference cat)			
	yes/ sometimes	0.46	0.18	**0.013**
Nationality	Greek (reference cat)			
	Other	−0.57	0.21	**0.010**
(CES-D)		− 0.01	0.01	**0.027**
Multiple linear regression (stepwise method) with dependent variable the Physical Component Summary (PCS) and independent variables demographic characteristics and CES-D scale (*N* = 90)				
Age		−0.29	0.07	**< 0.001**
Co-morbidity	no (reference cat)			
	Yes	−6.60	2.47	**0.009**
CES-D		−0.23	0.09	**0.010**
Multiple linear regression (stepwise method) with dependent variable the Mental Component Summary (MCS) and independent variables demographic characteristics and CES-D scale(*N* = 90)				
Age		−0.09	0.04	**0.017**
Physical activity	low (reference cat)			
	Medium	3.78	1.68	**0.027**
	High	3.73	2.59	0.154
CES-D		−0.55	0.07	**< 0.001**

^a^correlation coefficient ^b^standard error

Multiple linear regression (stepwise method) with dependent variable the Physical Component Summary (PCS) and independent variables demographic characteristics and CES-D scale revealed that age, co-morbidity, and depression score were found to correlate independently with Physical Component Summary. More specifically older participants scored lower in Physical Component Summary, Participants with co-morbidity scored 6.60 units lower than those with no co-morbidity according to the Physical Component Summary. Participants with more depressive symptoms their physical health is deteriorating.

Multiple linear regression (stepwise method) with dependent variable the Mental Component Summary (MCS) and independent variables the demographic characteristics and the CES-D scale, revealed that age, physical activity and depression score were found to correlate independently with Mental Component Summary (Table 2). More specifically older participants scored lower in Mental Component Summary. Participants with medium physical activity scored 3.78 units higher in Mental Component Summary, hence they present better mental health compared with participants with low activity. Participants with more depressive symptoms present an overall worsening of their mental health.

Physical functioning was evaluated in the lower mean score (40.6), while bodily pain in the higher score (74). SF – 36 and PACIC+ scale scores (Table 3) show a positive and low correlation in several subscales. The total score of PACIC+ scale as well as the Patient activation score correlate significantly with vitality. More specifically both scores increased in higher scores of vitality (*p* = 0.034 & *p* = 0.028 respectively). Delivery System / Practice Design score was increased in higher scores of mental health (*p* = 0.01) and MCS (*p* = 0.03). Follow-up/ Coordination was increased in higher scores of bodily pain (*p* = 0.01), vitality (*p* = 0.002), social functioning (*p* = 0.028), mental health (*p* = 0.024) and MCS (*p* = 0.004).

**Table 3:** SF – 36 and PACIC+ scale scoring (*N* = 90)

	PACIC+ summary		Patient activation	Delivery System design/Decision support	Goal Setting	Problem Solving/ Contextual counselling	Follow-up/ Coordination
Physical functioning	r	0.07	0.12	−0.09	0.02	0.08	0.12
	p	0.508	0.255	0.429	0.877	0.443	0.276
Role Physical	r	0.01	0.04	−0.04	−0.03	0.01	0.05
	p	0.923	0.708	0.743	0.765	0.940	0.621
Bodily pain	r	0.11	0.09	−0.06	0.06	0.01	0.27
	p	0.322	0.394	0.597	0.550	0.945	**0.010**
General health	r	0.20	0.17	0.14	0.13	0.18	0.19
	p	0.064	0.118	0.204	0.235	0.087	0.070
Vitality	r	0.23	0.23	0.10	0.08	0.18	0.32
	p	**0.034**	**0.028**	0.358	0.466	0.088	**0.002**
Social functioning	r	0.20	0.14	0.12	0.12	0.17	0.23
	p	0.064	0.202	0.278	0.246	0.104	**0.028**
Role Emotional	r	0.01	0.01	−0.06	−0.05	−0.01	0.13
	p	0.929	0.941	0.560	0.624	0.896	0.222
Mental health	r	0.19	0.16	0.27	0.07	0.11	0.24
	p	0.069	0.137	**0.010**	0.517	0.297	**0.024**
PCS	r	0.12	0.09	−0.04	0.10	0.13	0.15
	p	0.280	0.402	0.749	0.396	0.240	0.175
MCS	r	0.20	0.11	0.24	0.03	0.14	0.31
	p	0.075	0.327	**0.030**	0.766	0.202	**0.004**

Depression score (CES – D scale) was negatively correlated with several PACIC+ scale scores (Table 4). Higher depression mean score lead to lower PACIC+ summary score (*p* = 0.012), patient activation score (*p* = 0.046), Delivery System / Practice Design score (*p* = 0.036) and mean Follow-up/ Coordination score (*p* = 0.001).

**Table 4:** PACIC+ and CES – D (*N* = 90)

		CES-D
PACIC+ summary	r	−0.27
	p	**0.012**
Patient activation	r	−0.21
	p	**0.046**
Delivery System Design/ Decision Support	r	−0.23
	p	**0.035**
Goal Setting	r	−0.16
	p	0.142
Problem Solving/ Contextual counselling	r	−0.15
	p	0.170
Follow-up/ Coordination	r	−0.36
	p	**0.001**

## Discussion

Our findings demonstrate that almost 40% of our chronic diabetes patients co-suffered with hypertension, in agreement to previous studies which proved diabetes was associated with an increased risk of vascular complications, which leads to a decrease in QoL (eg hypertension, blindness, renal insufficiency / need for hemodialysis, limb rash, limb amputation, etc.) [49–55]. This study also revealed that one third of the participants suffered from mild depression, in agreement with previous findings of relevant studies, which reported important mental and emotional effects of diabetes patients [10] and strong correlation between symptoms of depression and diabetes [8, 56–60]. Cramm & Nieboer [24] have presented more often occurring depressive symptoms are associated with lower PACIC+ scores.

In the study at hand eating habits, nationality and depression scores were found to correlate independently with PACIC+ score. Glasgow and colleagues were not able to demonstrate significant differences in PACIC+ scores regarding patients’ sociodemographic characteristics within their particular research setting in the United State of America [20, 21]. However, Rosemann and colleagues managed to identify significant differences on the basis of age, education and psychiatric symptoms in specific European health care facilities [25].

In Cramm’s and Nieboer’s study there were specific categories of the sample, such as the young and less depressed patients which aligned with higher PACIC+ scores, and therefore verifying that their care is closely associated with the CCM [24]. However, the two studies (Cramm-Nieboer and Rosemann and colleagues) differ on how education is related to PACIC+ scores. On the one hand Rosemann and colleagues’ findings present a significant relation between education and PACIC+ scores, while Cramm’s and Nieboer’s study showed no significant relation exists between the aforementioned. This difference may be explained by the duration of the disease. Unlike Rosemann’s and colleagues study in which patients had been suffering from osteoarthritis for over 14 years, Cramm’s and Nieboer’s patients were only recently diagnosed [24, 29]*.*

The finding that younger and less distressed patients are more likely to report high PACIC+ scores may also indicate differences in physicians’ actions towards different groups of patients, and that these patients pursue CCM-compliant treatment more aggressively. However, this correlation is non-conclusive. However, this specific insight is useful in any case, because it implies that ensuring that all groups of patients benefit equally from any improvements in treating chronic diseases is an important feature in implementing CCM [24].

In addition this study showed that participants who followed a balanced diet experienced a better overall care than those who did not. They also received better care with regard to the dimensions of patient activation, the design of the decision support system, as well as monitoring, coordination and feedback.

Mean values of subscales of the PACIC+ questionnaire show that participants reported relatively moderate levels of satisfaction for the diabetes care. The lowest mean value was found in the goal setting subscale, which was slightly above the middle of the scale, followed by the patient’s activation subscale. The mean values of all subscales varied to similar levels to a previous conducted survey in Colorado on 363 Type II diabetic patients, as the target setting subscale received the lowest score and was related to the monitoring, coordination and feedback subscale, suggesting that they were almost similar to the Model of Chronic Care [21], which varied at a relatively modest level. This could be explained by the fact that health care systems had already included model elements in their day-to-day clinical practice through choice of approach and will or by the specificity of diabetic patients which comply more to the Chronic Care Model [61], with demands in their care for both dietary habits and education for medication.

Therefore, care provided to diabetic patients should take into account their need to be aware of the clear goals of the treatment. It should also involve them in the healing process, which will contribute to their further activation. Monitoring of care, coordination and feedback, reflect the communication deficit and management of the disease. There is a problem-solving subscale with specialized counseling which should be taken seriously into account by specialists taking care of DM patients.

Finally, the highest mean value was received by the Delivery System design/Decision support of the care system, which means that despite the above results patients still believe that caring system effectively supports them. Several studies have occasionally addressed satisfaction with the treatment of diabetic patients [52–54] in order to finally fulfill the ultimate goal of improving the quality of the treatment provided.

Our research revealed lowest mean value for the dimensions of SF-36, referring to the physical role and general health. Interestingly, the mean value of PCS score was lower than the mean of the corresponding MCS, suggesting that the participants experienced more physical than mental burden on their health. However, mental and physical health values remain at low levels that require improvement. In our study, age, suffering from other disease and depression score, were found to correlate independently with Physical Component Summary, while age, physical activity and depression with the Mental Component Summary. Diabetes impacts QoL immensely [62, 63] and has intense effects on the incidence of depression [64], a rather important feature of comorbidity in most chronic diseases. Several studies have presented the negative effects of depression on a patient’s ability to fulfil its self-management skills [65] and therefore depression may as well contribute to a rather poor attitude towards his/her efforts to glycaemic control [65, 66].

In addition, our study results showed that there have been significant positive correlations between several dimensions of care received and QoL, which agree with the main positions of the Chronic Care Model. Specifically, more vitality was correlated with more care and patient activation. Furthermore, higher mental health rates were correlated with considerably higher rates in the design of the care system with decision support, and higher values ​​in the dimensions of physical pain, vitality, social role and mental health were correlated with higher values ​​in the care dimension that includes monitoring, coordination and feedback. Findings from other studies are controversial with Cramm & Nieboer [24] suggesting that QoL could not be predicted by PACIC+ ratings and implying that the severity of the chronic disorder itself cannot be control by the care provided to chronically ill patients, However, in a study from Great Britain, lower scores of SF-36 were associated with the greater burden of diabetes caring [67]. Results from another study about improving care for diabetes patients indicated that SF36’s Physical Component Summary (PCS) and Mental Component Summary (MCS) scores for diabetes patients were substantially lower than the diabetes-free individuals [68].

In the limitations of the study it should be mentioned that the sample comes from a single healthcare service so we cannot claim to have a representation of all Greek healthcare organizations. In addition, the findings of this study are limited to a population that resides in a particular geographical area and refers to a disease with perhaps different approaches in terms of treatment. Another limitation could be the use of SF-36 as a measure of Qol and not a diabetes-specific measure, such as ADDQoL and DQoL and ADS which were not available and issues in terms of feasibility, complexity and length make them unattractive for use.

## Conclusions

This study presented important insights to the DM patients’ perception of the health services they receive. It concluded that the care provided to diabetic patients should take into account their need to be aware of the clear goals of the treatment. That is why patients should be involved more closely in the healing process, which will contribute to their further activation in the management of the disease. Despite the fact that DM patients believe that the caring system effectively supports them, the design of Delivery System /Decision support of the care system, should be improved.

## Data Availability

The datasets generated and analyzed during the current study are not publicly available to preserve the privacy of the participants but are available from the corresponding author on reasonable request.

## Abbreviations

PACIC+: Patient Assessment of Chronic Illness Care plus
PACIC: Patient Assessment of Chronic Illness Care
SF-36: The Short Form (36) Health Survey
QoL: Quality of life
DDQoL: Diabetes-dependent QoL
DQoL: Diabetes QoL
ADS: Appraisal of Diabetes Scale
CES-D: Center for Epidemiologic Studies Depression Scale
SPSS: Statistical Package for the Social Sciences
DM: Diabetes mellitus
SD: Standard deviation
HbA1c: Glycated hemoglobin
HRQL: Health Related QoL
PF: Physical functioning
RP: Role physical
BP: Bodily pain
GH: General health
VT: Vitality
SF: Social functioning
RE: Role emotional
MH: Mental health
CCM: The Chronic Care Model
WHO: World Health Organization
PCS: Physical Components Summary
MCS: Mental Components Summary
